# Paxlovid (Nirmatrelvir and Ritonavir) Use in Pregnant and Lactating Woman: Current Evidence and Practice Guidelines—A Scoping Review

**DOI:** 10.3390/vaccines11010107

**Published:** 2023-01-01

**Authors:** Prabal Chourasia, Babu Sriram Maringanti, Morgan Edwards-Fligner, Karthik Gangu, Aniesh Bobba, Abu Baker Sheikh, Rahul Shekhar

**Affiliations:** 1 Department of Hospital Medicine, Mary Washington Hospital, Fredericksburg, VA 22401, USA; 2 Department of Internal Medicine, University of New Mexico Health Sciences Center, Albuquerque, NM 87106, USA; 3 Department of Internal Medicine, University of Kansas Medical Center, Kansas City, KS 66160, USA; 4 Department of Medicine, John H Stronger Hospital, Cook County, Chicago, IL 60612, USA

**Keywords:** paxlovid, pregnancy, breast feeding, COVID-19

## Abstract

COVID-19 virus, since the detection of the first case in Wuhan in 2019, has caused a worldwide pandemic with significant human, economic and social costs. Fortunately, several vaccines and treatments, both IV and oral, are currently approved against the COVID-19 virus. Paxlovid is an oral treatment option for patients with mild-to-moderate disease, and it effectively reduces disease severity in high-risk patients. Paxlovid is an oral antiviral that consists of a combination of nirmatrelvir and ritonavi. As an oral medication suitable for outpatient treatment, it reduces the cost, hospitalization and mortality associated with COVID-19 infection. The pregnant population is a high-risk category for COVID-19 disease. Given their exclusion in clinical trials, there is limited data regarding Paxlovid use in pregnant and lactating women. Indirect evidence from ritonavir use as part of HAART therapy in the pregnant and lactating population with HIV has shown no significant teratogenicity. Moreover, animal studies on the use of nirmatrelvir do not suggest teratogenicity. This article summarizes the available data on ritonavir and nirmatrelvir use during pregnancy and in ongoing clinical trials. We also review the recommendations of major societies worldwide regarding Paxlovid use in pregnant and breastfeeding patients.

## 1. Introduction

COVID-19 infection, since the detection of the first case in Wuhan in 2019, has caused a worldwide pandemic with significant human, economic and social costs [1]. The emergence of new variants, with their ability to evade vaccine immunological protection, has made it difficult to contain the virus with vaccination only [2,3]. Moreover, vaccination hesitancy worldwide has made it challenging to control the morbidity and mortality associated with the COVID-19 pandemic [4,5]. 

Certain high-risk groups, such as pregnant females, are at increased risk of poor outcomes from acute COVID-19 infection [6,7]. Ellington et al. found that being pregnant was associated with a significantly higher risk of hospitalization (relative risk, 0.9; 95% confidence interval 0.5–1.5) in their study of a cohort of 91,412 women, which included 8207 pregnant women [8]. Further increasing the risk in pregnant females could be the lower vaccination acceptance rate in women, as noted by Troiano et al. in their narrative review of 15 studies [5]. However, the transmission of SARS-COV-2 through breast milk is considered uncommon [9]. Some pregnant and breastfeeding females may not be eligible for vaccination due to side effects and contraindications [10].

Data on the safety of remdesivir use during pregnancy are inconclusive, and its use is based on assessing the benefit–risk profile [11]. Antiviral medications, such as Paxlovid, can add an effective armament in the fight against COVID-19, but there are conflicting recommendations from medical societies worldwide about its use during pregnancy. Given the important role that Paxlovid can play in improving the outcomes of COVID-19 infection and the limited data on its safety during pregnancy, a thorough review of the existing evidence and expert opinions is needed to understand its safe use during pregnancy. 

## 2. Materials and Methods

Two reviewers (Maringanti. S and Chourasia. P) performed a preliminary search in “PUBMED”, “EMBASE”, “WEB OF SCIENCE” and NIH COVID-19 portfolios, and medRxiv was also searched. Any disagreements between the reviewers were resolved by a third reviewer (Baker. A). Title and abstract screening and full-text screening with the interpretation of the study results were carried out using specific inclusion and exclusion criteria. We included articles published between 1 January 2020 and 20 August 2022. Only those articles that were either originally in English or translated into English were included. The search terms used reflected key features of the review question. These terms included “paxlovid”, “nirmatrelvir and ritonavir”, “pregnancy” and “lactation or breast feeding”. We included retrospective descriptive studies, randomized controlled trials, cohort studies and case–control studies. Lastly, peer-reviewed published papers and non-peer-reviewed preprints were incorporated into the review.

## 3. Results

### 3.1. Paxlovid for COVID-19 

Paxlovid is an oral antiviral that combines nirmatrelvir and ritonavir [12]. In December 2021, the FDA approved Paxlovid for patients with mild or moderate COVID-19 in the outpatient setting who are at risk of progression to severe disease [13]. The initial clinical trial data support its utility in reducing the risk of progression to severe illness and hospitalization [14]. As an oral medication used in an outpatient setting, it can reduce the overall cost by reducing the hospitalization, morbidity and mortality associated with COVID-19 infection [15]. 

Though current and recent pregnancy is an important factor that can increase the risk of severe COVID-19 disease [6,16], phase 2/3 clinical trials for nirmatrelvir/ritonavir in non-hospitalized patients (EPIC-HR, EPIC-SR and EPIC-PEP) excluded pregnant and breastfeeding individuals [14,17]. The EPIC-HR trial evaluated protease inhibition for COVID-19 in high-risk patients, the EPIC-SR trial evaluated protease inhibition for COVID-19 in standard-risk patients, and the EPIC-PEP trial evaluated protease inhibition for COVID-19 in post-exposure prophylaxis. While Paxlovid can be an important tool to reduce the adverse outcomes associated with COVID-19 disease during pregnancy, human data regarding its safety during pregnancy are limited.

### 3.2. Mechanism of Action of Paxlovid

Paxlovid has two components: nirmatrelvir and ritonavir. Nirmatrelvir is an oral protease inhibitor active against the SARS-COV-2 3-chymotrypsin-like cysteine protease enzyme (Mpro) [18]. Mpro is an important part of viral replication, and it does not have recognized human analogues [19,20]. This makes it an ideal target against the SARS-COV-2 virus [20]. In a mouse model, its oral administration was associated with lower SARS-COV-2 titers compared to the placebo [18]. Nirmatrelvir is metabolized primarily by CYP3A4 [18,21]. Ritonavir is a strong P450 3A4 inhibitor and is commonly used as part of antiretroviral therapy for the treatment of Human Immunodeficiency Virus (HIV) infection. It increases the bioavailability of nirmatrelvir to target the therapeutic range [21,22]. 

### 3.3. Safety and Adverse Effects of Paxlovid

Nirmatrelvir (300 mg) and ritonavir (100 mg) in twice-daily doses were noted in a simulation to maintain plasma trough concentration 5–6 times in vitro 90% effective concentration (based on data from the first human study in healthy participants) [14,23]. 

The EPIC-HR trial studied the safety and efficacy of Paxlovid in patients with COVID-19 infection who were at increased risk of developing severe illness but did not need to be hospitalized [14]. This was a phase 2/3 double-blind, randomized, controlled trial with 2246 participants (symptomatic, unvaccinated, non-hospitalized adults with high-risk features) and contained a 1:1 drug and placebo group. The common adverse events were mainly graded 1 (denoting mild severity) and resolved after completing the treatment. These adverse events included dysgeusia (5.6% vs. 0.3%), diarrhea (3.15% vs. 1.6%), headache (1.4% vs. 1.3%) and vomiting (1.1% vs. 0.8%) when comparing Paxlovid vs. placebo. Patients receiving Paxlovid had lesser grade 3 or 4 adverse events than placebo recipients (4.1% vs. 8.3%), had fewer adverse events (1.6% vs. 6.6%) and had fewer adverse events requiring drug stoppage (2.15 vs. 4.2%). In this study, there were 13 deaths, all among placebo recipients. In a University of Washington study post-FDA approval (total patients=50, carried out through telephonic post-prescription outreach using a standardized questionnaire), the most commonly reported adverse effects were dysgeusia (57.5%), diarrhea (37.5%), headache (32.5%), abdominal pain (15%), nausea (12.5%) and vomiting (10%) [24]. A total of 85% of patients reported at least one side effect, and 30% reported three or more adverse effects. The onset of most symptoms was within 1–2 h of taking Paxlovid and resolved within 24 h of stopping the medication. Zheng et al. also found Paxlovid use to be safe and effective for the approved population in a meta-analysis of 13 studies [25]. 

Of note, these studies excluded an important high-risk group for acute COVID-19 infection, e.g., pregnant and breastfeeding patients, necessitating this scoping review.

### 3.4. Review of Paxlovid Data in Pregnant and Lactating Woman 

Clinical trials investigating Paxlovid use in patients with mild-to-moderate COVID-19 with a high risk of progression to severe diseases have excluded pregnant and lactating women. Paxlovid is being prescribed on a case-by-case basis under emergency use authorization in the USA after obtaining patient consent. Evidence is limited regarding Paxlovid use in pregnant and lactating women, with no completed clinical trial in this population.

### 3.5. Human Studies of Paxlovid

Loza et al. conducted a retrospective descriptive study of seven pregnant patients who received nirmatrelvir/ritonavir therapy and experienced symptom resolution without immediate adverse effects. No adverse fetal or neonatal effects were observed [26]. Out of the seven pregnant patients, one patient was induced at 37 2/7 weeks for cholestasis of pregnancy, one spontaneously delivered a healthy neonate at 40 3/7 weeks and one opted for an elective induction at 39 1/7 weeks and delivered a healthy neonate. The remaining four patients were still pregnant. This study had several limitations, including being descriptive, having a low number of patients, and having no long-term maternal and fetal follow-up.

### 3.6. Human Studies of Ritonavir

Since there is no direct evidence of Paxlovid use in pregnant and lactating women, we reviewed the evidence regarding individual drug use. There are extensive data on the safety of ritonavir use because of its use as part of antiretroviral therapy (ARV) for pregnant women infected with HIV. There is potential for confounding while assessing data regarding the risk and side effects of ritonavir use in pregnant patients infected with HIV. However, given the limited available data on Paxlovid to date, it may help to understand ritonavir’s overall side effect profile based on extensive prior use in pregnant patients infected with HIV. Sibiude et al. performed an in-depth analysis of a sub-study of a French perinatal cohort of women starting protease-inhibitor (PI)-based ARV therapy during pregnancy (*n* = 1253) from 1990 to 2009 in order to evaluate trends in prematurity (<37 gestational weeks) [27]. During the period of 2005–2009, the prematurity rate was higher with boosted than with non-boosted protease inhibitor therapy started during pregnancy (14.4% vs. 9.1% [*p* = 0.05]; adjusted hazard ratio, 2.03 [95% CI, 1.06–3.89; *p* = 0.03] in a multivariate analysis). The difference was noted primarily in induced preterm delivery for maternal or fetal indications (5.6% vs. 1.6%; *p* = 0.02). The prematurity rate among pregnant women infected with HIV was twice that of the general population; sociodemographic characteristics did not entirely explain this result. Prematurity was independently associated with antiretroviral therapy, particularly with the initiation of ritonavir-boosted PI therapy during pregnancy. 

Pasley et al. performed a systematic review of the safety and efficacy of lopinavir/ ritonavir-based antiretroviral therapy in 2675 pregnant women infected with HIV (including HIV-1 RNA at delivery). This review suggested no unique safety or efficacy concerns with standard-dose lopinavir/ritonavir-based antiretroviral therapy in pregnant women [28].

Kakkar et al. studied the association between preterm birth, antiretroviral drug exposure and maternal risk factors retrospectively using logistic regression in 705 women who tested positive for HIV [29]. Among the women treated with antiretroviral therapy, the risk of preterm birth was significantly higher among the women who received boosted versus non-boosted protease inhibitors (OR 2.01, 95% CI 1.02–3.97). This remained significant even after adjusting for maternal age, delivery CD4 count, hepatitis C co-infection, history of previous preterm births and parity (aOR 2.17, 95% CI 1.05–4.51). The results indicated the possible role of ritonavir boosters as a risk factor for preterm birth.

Sibiude et al. studied the risk of liver enzyme elevation in pregnant women receiving protease inhibitors through a retrospective analysis of pregnant women taking antiretroviral therapy who had >1 times the normal measure of liver enzymes during pregnancy, and they concluded that there is a possible role of protease inhibitors in liver enzyme elevations [30].

Beckerman et al. studied low birth weight (LBW) in the Antiretroviral Pregnancy Registry through a retrospective study of pregnant patients with HIV on antiretroviral therapy involving 11,105 live births [31]. The study showed an increased risk of low birth weight after maternal protease inhibitor exposure compared to combination antiretroviral therapy without a protease inhibitor. This increase persisted after adjusting for low-birth-weight risk factors in multivariate analyses. The presence or absence of ritonavir in PI-based combinations did not affect the low-birth-weight risk.

Favarato et al. examined data from the United Kingdom/Ireland National Study of HIV in Pregnancy and Childhood on women with HIV delivering a singleton live infant in 2007–2015, including those who were receiving ritonavir-boosted protease inhibitor-based (*n* = 4184) or non-nucleoside reverse transcriptase inhibitor (NNRTI)-based (*n* = 1889) regimens [32]. This study examined two groups, those with CD4 counts less than 350 cells per cubic millimeter of blood and those with CD4 counts greater than 350 cells per cubic millimeter of blood. The use of lopinavir/ritonavir was associated with increased preterm delivery risk vs. women on NNRTI-based regimens in both groups (odds ratio 1.99 (95% CI 1.02–3.85) in CD4 counts less than 350 group and odds ratio 1.61 (95% CI 1.07–2.43) for CD4 counts greater than 350 group). The study concluded that there is a link between the initiation of ritonavir-boosted/lopinavir-based antiretroviral therapy preconception and preterm birth in subsequent pregnancies.

Can et al. performed a retrospective study of 122 pregnant patients with COVID-19 who had elevated liver enzymes, and they found a possible association between lopinavir/ritonavir use and elevated liver enzymes [33].

Saint-Lary et al. conducted a retrospective study to evaluate the pregnancy outcomes from in utero exposure to antiretroviral drugs of 122 pregnant women infected with HIV who used antiretroviral treatment, including protease inhibitors. Of 918 deliveries (of which 88% had documented pregnancy outcomes), the results showed as the primary outcome that a protease inhibitor-based regimen is overall reassuring on the risk of adverse pregnancy outcomes [34].

### 3.7. Animal Studies of Nirmatrelvir

There is only one animal study on the reproductive and developmental safety of nirmatrelvir. Catlin et al. assessed the potential effects of nirmatrelvir on embryo–fetal development in rats and rabbits and on fertility and early embryonic development in rats with a limit dose of 1000 mg/kg/day [35]. This study did not note any severe developmental toxicity in rats or rabbits. Moreover, this study did not report any effects on rats’ early embryonic development and fertility.

The studies mentioned above have several limitations. Most of them are retrospective and do not have a long-term follow-up, which limits the validity of the results. We found only one human study of Paxlovid use during pregnancy, and it had major limitations; for example, the nature of the study was descriptive, and it had inadequate power and a lack of long-term follow-up. We did not find any data regarding the effects of nirmatrelvir or Paxlovid on breastfeeding. According to the National Institute of Health (NIH) database, ritonavir is excreted into milk in measurable concentrations, and low levels can be found in the blood of some infants. Still, the effects on breastfed infants are unknown [36]. Nirmatrelvir is a new drug whose long-term effects are not yet known. Moreover, there are no definitive data regarding Paxlovid’s effect on male and female fertility based on current human and animal studies.

Before prescribing Paxlovid, it is essential to review the patient’s concurrent medications due to several drug interactions attributed to the ritonavir component of Paxlovid. Ritonavir is a potent cytochrome P450 (CP) 3A4 inhibitor and a p-glycoprotein inhibitor. Ritonavir increases the effectiveness of nirmatrelvir by increasing its blood concentration. Similarly, ritonavir can increase the concentrations of various drugs in the blood and can lead to toxicity. 

Several of the commonly used medications during pregnancy can be safely continued without drug-related interactions. The National Institute of Health’s website has an extensive list of medications with recommendations on whether to continue or withhold the medication and whether to modify the dose or advice against Paxlovid use [37]. It also contains a drug interaction checker that physicians can use before prescribing Paxlovid [38].

Several ongoing trials are evaluating the use of Paxlovid in acute COVID-19, including in pregnant women with acute COVID-19 (Table 1). One of the trials (NCT05386472) is primarily enrolling pregnant patients in order to study the use of Paxlovid to prevent the progression to severe COVID-19 disease in this vulnerable population.

## 4. Recommendations for Healthcare Providers from Major International Societies 

On 22 December 2021, the FDA approved Paxlovid in the United States under emergency use authorization (FDA-EUA) for the treatment of mild-to-moderate COVID-19 in high-risk individuals aged 12 years or older with a weight of 40 kg or more within five days of symptom onset [39] (Table 2). The National Institute of Health (NIH) recommends offering Paxlovid to qualifying pregnancy patients based on the risk–benefit profile [37]. This risk–benefit assessment may include medical comorbidities, body mass index, vaccination history and risk factors for severe COVID-19 disease [37]. NIH does not recommend Paxlovid to be contraindicated during breastfeeding and recommends considering the benefit of breastfeeding, medication need and risk of exposure to the infant with potential risk from COVID-19 infection [37]. The Society of Maternal-Fetal Medicine in the United States supports its use in pregnant patients under FDA-EUA, with caution regarding its extensive drug–drug interactions. The Government of Canada recommends against taking Paxlovid during pregnancy unless a healthcare professional advises otherwise (Table 2). The Government of Canada also advises non-pregnant women to use contraception while taking Paxlovid and leaves the decision to healthcare professionals as to whether Paxlovid can be used while breastfeeding [40]. Paxlovid use is not recommended by medical societies in Europe, the United Kingdom, Germany, Australia and New Zealand (Table 2).

## 5. Discussion

Nirmatrelvir inhibits the SARS-CoV-2 main protease, preventing viral replication [18], and to achieve adequate therapeutic plasma concentrations, it requires co-administration with ritonavir, an HIV-1 protease inhibitor and a CYP3A inhibitor. Nirmatrelvir’s mechanism of action suggests a low risk of teratogenicity. Since ritonavir is an important, widely used antiviral medication against HIV, there are plenty of data regarding its effect during pregnancy. A recent study consisting of 34 retrospective cohort studies noted that protease-inhibitor-based antiretroviral therapy for HIV is associated with an increased risk of babies who are “small for gestational age” and “very small for gestational age” but not with an increased risk of “preterm birth” or other adverse fetal and perinatal outcomes [49]. Similar to other viral protease inhibitors used in HAART for HIV, ritonavir exhibits good selectivity for its intended viral target, with favorable in vivo and in vitro *toxicity* profiles [18].

In animal studies, nirmatrelvir administration was not found to be associated with any malformation, decreased embryo–fetal survival or increased maternal mortality [35]. It was noted to be associated with low birth weight in rabbits receiving doses ten times the expected exposure to nirmatrelvir in Paxlovid. This effect was not seen in rats receiving the same dose and exposure length. Overall, animal studies do not suggest an increased risk of adverse maternal or fetal mortality with nirmatrelvir. Other observational studies have also demonstrated the safety of ritonavir during pregnancy [28]. In contrast to existing toxicity data showing the safety of nirmatrelvir use during pregnancy, Molnupravir (another oral COVID-19 medication approved by the FDA) has a higher likelihood of teratogenicity. Its mechanism of action can lead to mutagenesis in viral RNA and off-target effects in human cells [50].

The data on protease inhibitors support the safety of the ritonavir component of Paxlovid during pregnancy but also corroborate the findings in the animal model reproductive toxicology study of nirmatrelvir. Moreover, the data on ritonavir are based on extended use in HIV, where it is taken throughout pregnancy. In contrast, Paxlovid is only used for five days, making its use for acute COVID-19 infection even safer.

The current guidelines of the NIH COVID-19 treatment panel, the American College of Obstetricians and Gynecologists (ACOG) and the Society for Maternal-Fetal Medicine support the cautious use of Paxlovid during pregnancy based on a risk–benefit assessment [51,52]. Shared decision making with patients and the consideration of patients’ preferences, along with risk factors, are essential. The current guidelines of US societies, such as the Society for Maternal-Fetal Medicine and the National Institute of Health, agree that this therapy should not be withheld from pregnant and breastfeeding patients at higher risk of severe disease. A careful review of current medications for all patients, especially those with comorbid conditions, for interactions with Paxlovid is an important key step in this decision-making process [53].

While clinical trials of Paxlovid, to date, have not included pregnant or breastfeeding subjects, multiple studies have been proposed to include this important population (Table 1). One of these studies is The RECOVERY trial, which aims to identify treatments beneficial to patients hospitalized with suspected or confirmed COVID-19 disease, and it includes pregnant and breastfeeding individuals in its nirmatrelvir/ritonavir arm [54]. The results of this study will provide important data regarding the safety of Paxlovid in pregnant and breastfeeding females.

## 6. Conclusions

In conclusion, while safety data regarding Paxlovid are lacking in direct clinical trials, data regarding its individual components from prior studies and animal model studies support its safe use during pregnancy. The authors believe that vaccinations are still the safest way to protect against severe COVID-19 infection, though there are challenges for universal vaccination, including vaccination hesitancy and availability in developing nations, which can be a limiting factor in some special populations, such as pregnant populations. Given the risk of morbidity, hospitalization and mortality associated with severe COVID-19 disease in females and fetuses, Paxlovid can provide an important option to reduce the risks associated with acute COVID-19 infection in at-risk and unvaccinated patients after careful consideration of the benefit and risks of each individual patient. Further studies are needed to assess its safety profile in pregnant and breastfeeding female patients.

## Figures and Tables

**Table 1:** Ongoing clinical trials on Paxlovid use that include pregnant and breastfeeding females.

NCT Number	Title	Status	Study Type
NCT05567952 https://clinicaltrials.gov/ct2/show/NCT05567952 (accessed on 26 November 2022).	A Study to Learn About a Repeat 5-Day Treatment With the Study Medicines (Called Nirmatrelvir/Ritonavir) in People 12 Years Old or Older With Return of COVID-19 Symptoms and SARS-CoV-2 Positivity After Finishing Treatment With Nirmatrelvir/Ritonavir	Recruiting	Interventional
NCT05487040 https://www.clinicaltrials.gov/ct2/show/NCT05487040 (accessed on 26 November 2022).	A Study to Measure the Amount of Study Medicine in Blood in Adult Participants With COVID-19 and Severe Kidney Disease	Recruiting	Interventional
NCT05438602 https://clinicaltrials.gov/ct2/show/NCT05438602 (accessed on 26 November 2022).	A Study to Learn About the Study Medicines (Called Nirmatrelvir/Ritonavir) in People 12 Years Old or Older With COVID-19 Who Are Immunocompromised	Recruiting	Interventional
NCT05387369 https://clinicaltrials.gov/ct2/show/NCT05387369 (accessed on 26 November 2022).	A Real World Study of Paxlovid for the Treatment of Hospitalized Patients Confirmed With COVID-19	Recruiting	Observational
NCT05386472 https://clinicaltrials.gov/ct2/show/NCT05386472 (accessed on 26 November 2022).	A Study to Learn About the Study Medicine (Called Nirmatrelvir/Ritonavir) in Pregnant Women With Mild or Moderate COVID-19	Recruiting	Interventional
NCT05386433 https://clinicaltrials.gov/ct2/show/NCT05386433 (accessed on 26 November 2022).	Paxlovid in the Treatment of COVID-19 Patients With Uremia	Not yet recruiting	Interventional
NCT05263908 https://clinicaltrials.gov/ct2/show/NCT05263908 (accessed on 26 November 2022).	General Investigation for PAXLOVID PACK	Recruiting	Observational
NCT04381936 https://clinicaltrials.gov/ct2/show/NCT04381936 (accessed on 26 November 2022).	Randomized Evaluation of COVID-19 Therapy	Recruiting	Interventional

**Table 2:** Current recommendation regarding Paxlovid use in pregnant and breastfeeding females from major international societies.

Society	Country	Pregnancy Recommendation	Breastfeeding Recommendation
Society for Maternal- Fetal Medicine [41]	USA	- Supports use in appropriate patients - Supports NIH COVID-19 treatment guidelines - Encourages shared decision making	- Supports use in appropriate patients - Encourages shared decision making
National Institute of Health [37]	USA	- Recommends offering Paxlovid to qualifying pregnancy patients based on risk–benefit profile - Risk–benefit assessment may include medical comorbidities, body mass index, vaccination history and risk factors for severe COVID-19 disease	- Does not recommend Paxlovid to be contraindicated during breastfeeding - Recommends considering benefit of breastfeeding, medication need, risk of exposure to infant with potential risk from COVID-19 infection
National Health Service [42]	UK	Does not recommend its use	Does not recommend during breastfeeding during treatment and 7 days after last dose
Department of Health and Aged Care [43]	Australia	Does not recommend its use	Does not recommend its use
Health Navigator [44]	New Zealand	Does not recommend Paxlovid for pregnancy	Does not recommend breastfeeding or trying to become pregnant while on Paxlovid until 7 days after the end of treatment
European Medicine Agency [45]	Europe	Does not recommend during pregnancy, in people who can become pregnant or in people who are not using contraception	Breastfeeding should be interrupted during treatment
The Central Drugs Standard Control Organization [46]	India	No recommendation	No recommendation available
National Medical Products Administration # [47]	China	No recommendation available	No recommendation available
Government of Canada [40]	Canada	Does not recommend taking Paxlovid during pregnancy unless healthcare professional advises otherwise	Leaves the decision to healthcare professionals regarding use of Paxlovid during breastfeeding
Federal Institute for Drugs and Medical Devices in Germany [48]	Germany	Refers to European Medicines Agency	Refers to European Medicines Agency

## Data Availability

Not applicable.
